# The NRAMP1 polymorphism as a risk factor for tuberculous spondylitis

**DOI:** 10.5704/MOJ.1303.012

**Published:** 2013-03

**Authors:** Bambang Tiksnadi, Herry Herman

**Affiliations:** Department of Orthopaedic Surgery and Traumatology, Padjadjaran State University, Bandung, Indonesia; Department of Orthopaedic Surgery and Traumatology, Padjadjaran State University, Bandung, Indonesia

## Abstract

In the present study, we analysed the association between the incidence of tuberculous spondylitis with the Natural Resistance Associated Macrophage Protein 1 (NRAMP1, also known as Solute Carrier Family 11a member1) polymorphism by studying the genetic segregation of this polymorphism and the incidence of the disease among members of the West Javanese population undergoing surgery for tuberculous spondylitis at our institution. We compared the distribution of NRAMP1 polymorphism at two specific sites, namely D543N, and 3’UTR, among subjects with pulmonary tuberculosis and tuberculous spondylitis. We found no significant differences in distribution of polymorphism between the two groups, or between pulmonary tuberculosis and tuberculous spondylitis compared to healthy subjects. However, a pattern emerged in that polymorphisms at the two sites seemed to be protective against development of tuberculous spondylitis in our study population. We concluded that in the West Javanese population, there is no association between NRAMP1 polymorphism with the propensity for development of pulmonary tuberculosis or tuberculous spondylitis. In fact, NRAMP1 may provide protection against the development of tuberculous spondylitis.

**Key Words:**

tuberculous spondylitis, NRAMP1, polymorphism

## Introduction

Tuberculous infection (TB) may result in pulmonary or extrapulmonary complications. One of the more devastating extrapulmonary manifestations is tuberculous spondylitis (STB). At least half of tuberculous musculoskeletal infections eventually present as spondylitis. At our institution STB accounts for 40% of all patients requiring surgery for back problems. Among the most intriguing questions are what factors affect the natural course of the infection, whether it will first present as pulmonary tuberculosis (PTB), or as STB or as both at the same time.

Among the many types of Gell and Coombs hypersensitivity reactions, immunity against TB is mediated by delayed hypersensitivity. Since TB bacteria are intracellular, it takes some time for the individuals’ immune system to form immunity. Clearance of intracellular parasites is not mediated by an adaptive response, since mycobacterium antigens aren’t at the infected cell surface; instead, immunity arises in the form of macrophage aggregation under the influence of T-helper type 1 (Th1) cytokines, which in turn drive cellular responses. This results in walling-off of the infections by macrophages and monocytes cells as granulomas. Clearance of intracellular parasites is achieved by lysis of the infected cells and bacteria through the oxygen-dependent and oxygen-independent mechanisms. Among these mechanisms are the nitric oxide (NO) system that generates oxygen radicals and lysozymes in phagolysosomes that disintegrate parasites. Both of these mechanisms depend upon the transport of soluble factors such as Mn^2+^ and Fe^2+^ acting as reaction cofactors and acidity (pH) regulators^1^. One of the proteins facilitating such transport in macrophages, Natural Resistance Associated Macrophage Protein 1 (NRAMP1), is widely studied for its effect on TB outcomes. Due to its function in regulating influx of soluble factors it also is known by an alternate name, Solute Carrier Family 11a member^1^.

In an initial study in a West African population by Bellamy et. al., polymorphisms were identified at the 3’UTR, D543N, INT4 and 5’CA sites of the gene that correspond with susceptibility to PTB 2. Further genetics studies validated these results in closely related African regions^2^-^5^, but on the other hand, subsequent studies in Asian populations varied^6^-^10^, validated those results only at only certain sites, mostly at d543 and 3’UTR^11^,^12^. Several other studies including one in Sulawesi showed no correlation between polymorphism at NRAMP1 with susceptibility to PTB^13^. Results of animal experiments investigating the role of NRAMP1 in resistance to TB infection were inconsistent^14^.

Studies about the association between NRAMP1 polymorphism and extrapulmonary manifestation are limited, and most concentrated only on pleural manifestations^15^. A particular study about STB in India^16^ reported no significant association between NRAMP1 polymorphisms and STB. However, they did found increased polymorphisms at 3’UTR and D543N in STB.

The above results are intriguing; thus we sought to produce data on patients at our institution undergoing surgery for STB. Advances in knowledge about the polymorphism that are associated with STB may contribute improved identification of susceptible individuals and lead to improvements disease control and prevention in the future.

## Materials and Methods

The study sample consisted of patients who underwent surgery for STB for various indications between January 2003 and August 2008, and also included pulmonary TB patients and healthy study participants as controls.

**Figure 1:**
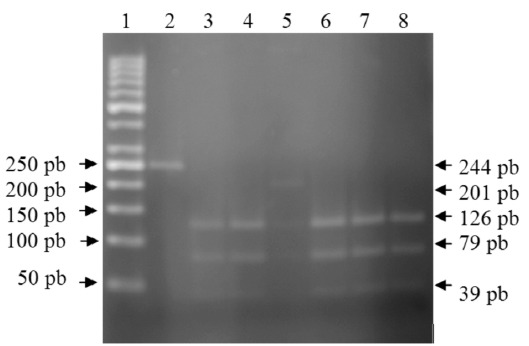
: PRepresentative RFLP (restriction fragment length polymorphism) profile of D543N with AvaII restriction. Lane 1, molecular size marker; lane 2, undigested product; lane 3,4,6,7,8 digested PCR fragments of 126 pb, 79 pb, 39 pb representing genotype GG, lane 5 digested PCR fragment of 201 pb, 126 pb, 79 pb, and 39 pb representing genotype GA.

**Figure 2:**
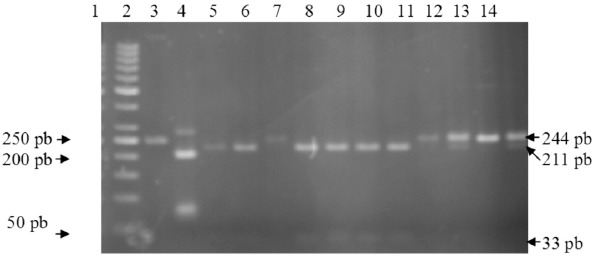
: Representative RFLP (restriction fragment length polymorphism) profile of 3’UTR with FokI restriction. Lane1,molecular weight marker 50; lane 2, undigested PCR product; lane 4,5,7,8,9,10 digested PCR fragments of 211 and 33 pb representing TGTG+/TGTG+ genotype. Lane 6,11,13 digested PCR fragment of 244 pb (not fragmented) representing TGTG-/TGTG-genotype. Lane 12,14. Digested pcr fragments of 244 pb,211 pb, and 33 pb representing TGTG+/TGTG- genotype.

Polymorphism at D543N and 3' UTR sites were identified using published primers and the restriction fragment length polymorphism (RFLP) method. Briefly, DNA was prepared from blood utilizing a high purity polymerase chain reaction (PCR) template preparation kit (Boehringer Mannheim, Mannheim, Germany); we used PCR and PCR RFLP utilized primers: F 5’-gca tct ccc caa ttc atg gt-3’ and R 5’ aac-tgtccc- act-cta-tcc-tg 3’ for D543N and F 5’gca-tct-ccc-caa-ttcatg- gt 3’ and R 5’tgt- ccc-act cta-tcc-tg-3’ for 3’UTR polymorphism (Bbioneer Kaist, Taejon, Republic of Korea). AvaII restriction was used to differentiate between D543N polymorphisms (Figure 1), and FokI was utilized to differentiate between 5’UTR polymorphisms (Figure 2). The distribution of polymorphisms at both sites was compared in STB and PTB participants and between STB and healthy participants. Chi square analysis was used to assess statistical differences between the distributions of polymorphisms utilizing commercial statistical analysis package SPSS version 10 for Windows (SPSS Inc., Chicago, Il, USA).

The ethics committee of the Hasan Sadikin Hospital approved the study protocol and the study was conducted in accordance with the Helsinki Declaration (1975).

## Results & Discussion 

Forty-one STB patients (26 (63.4%) females and 15 (36.6%) males) of West Javanese descent domiciled in and around the city of Bandung (71.4%) as well as from various outlying area were recruited for this study (Table I; mean age, 35.9 ± 11.3 y (range, 15- 66 y. Ninety per cent of these patients had back pain, 76% had gibbus, and 54% had neurological deficit. One hundred sixty four patients with Pulmonary Tuberculosis and 123 healthy subjects were included as controls resulting in a total of 328 study participants (Table I).

We found no significant differences in polymorphism distribution between STB and PTB at D543N (p=0.56) or at 3’UTR (p=0.40) (Table II). We therefore believe that the polymorphisms have no decisive role in determining the natural outcomes of TB infection (i.e., PTB or STB). This result is more aligned with recent studies on Asian population, showing that putative race and ethnic background supercede the influence of NRAMP1 on TB outcomes.

Further, we found no significant differences in polymorphism distribution between STB and healthy subjects, either at D543N (p=0.92) nor at 3’UTR (p=0.92) (Table II), leading us to believe that that the polymorphism does not have a role in increased susceptibility to STB in our population. This finding was similar to results reported by Selvaraj et al.^16^

Since about half of the study population in the STB group also had PTB, we attempted to investigate the role of the NRAMP1 polymorphism in increasing susceptibility of PTB patients to STB. To do so, we compared the polymorphism distribution in the PTB only group to the STB+PTB group. Although, we found no significant distribution differences at D543N (p=0.295) or at 3’UTR (p=0.234) (Table III), there were increasing polymorphisms at both sites in subjects with PTB only, compared to subjects with STB+PTB, perhaps indicating that the polymorphism is instead associated with protection against STB for PTB patients. This result contradicts the results of in India by Selvaraj et. al.^16^ querying the same sites. Possible explanations may lie in the fact that our study compared the polymorphism distribution differences between cases of PTB and PTB + STB, while the study in India compared STB to normal healthy controls. Taken together, however, the results point to an effect on the propensity for development of STB. Definitive answers lie in a future, larger epidemiological study.

**Table I:**
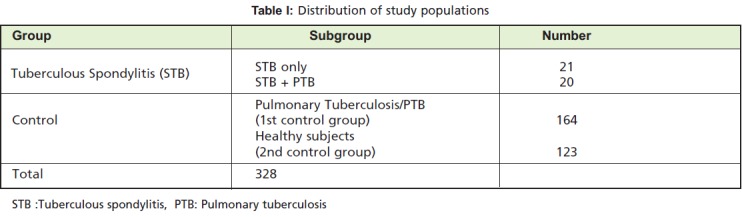
: Distribution of study populations

**Table II:**
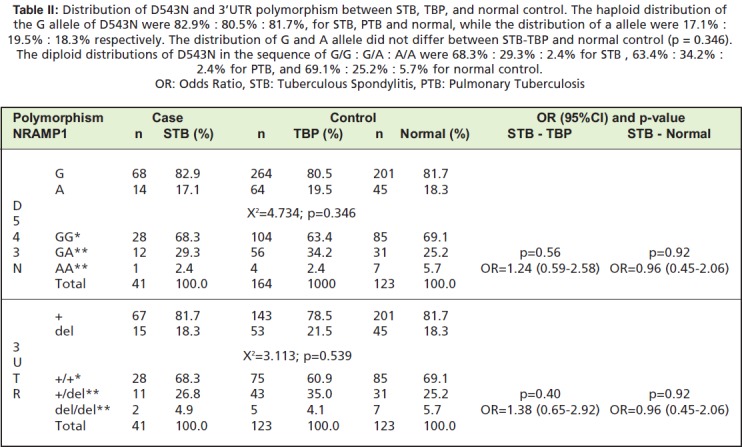
: Distribution of D543N and 3’UTR polymorphism between STB, TBP, and normal control. The haploid distribution of the G allele of D543N were 82.9% : 80.5% : 81.7%, for STB, PTB and normal, while the distribution of a allele were 17.1% : 19.5% : 18.3% respectively. The distribution of G and A allele did not differ between STB-TBP and normal control (p = 0.346). The diploid distributions of D543N in the sequence of G/G : G/A : A/A were 68.3% : 29.3% : 2.4% for STB , 63.4% : 34.2% : 2.4% for PTB, and 69.1% : 25.2% : 5.7% for normal control. OR: Odds Ratio, STB: Tuberculous Spondylitis, PTB: Pulmonary Tuberculosis

**Table III:**
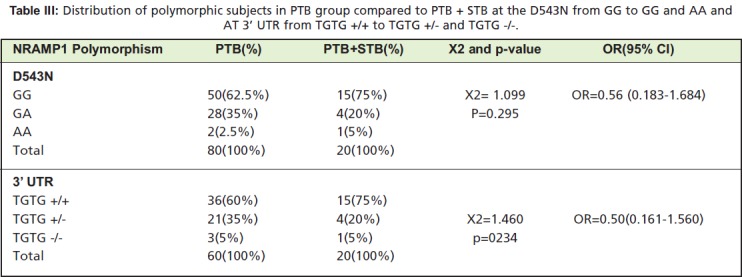
: Distribution of polymorphic subjects in PTB group compared to PTB + STB at the D543N from GG to GG and AA and AT 3’ UTR from TGTG +/+ to TGTG +/- and TGTG -/-.

## Conclusion

We conclude that in West Javanese patients undergoing surgery for STB in our institution, there were no associations between NRAMP1 polymorphisms at D543N and 3’UTR sites and susceptibility to development of STB, PTB or STB+ PTB. Of note, within the PTB group, we found that polymorphisms provide a certain level of protection against development of STB+ PTB. Further study with a larger sample is warranted to produce a statistically relevant observation.

## References

[1] McDermid JM, Prentice AM (2006). Iron and infection: effects of host iron status and the iron-regulatory genes haptoglobin and
NRAMP1 (SLC11A1) on host-pathogen interactions in tuberculosis and HIV. Clin Sci.

[2] Bellamy R, Ruwende C, Corrah T, McAdam KP, Whittle HC, Holl AV (1998). Variations in the NRAMP1 gene and susceptibility to
tuberculosis in West Africans. N Engl J Med.

[3] Cervino AC, Lakiss S, Sow O, Hill AV (2000). Allelic association between the NRAMP1 gene and susceptibility to tuberculosis in
Guinea-Conakry. Ann Hum Genet.

[4] El Baghdadi J, Remus N, Benslimane A, El Annas H, Chentoufi M, Abel L, Schurr E (2003). Variants of the human NRAMP1 gene and
susceptibility to tuberculosis in Morocco. Int J Tuberc Lung Dis.

[5] Hoal EG, Lewis LA, Jamieson SE, Tanzer F, Rossouw M, Victor T (2004). SLC11A1 (NRAMP1) but not SLC11A2 (NRAMP2)
polymorphisms are associated with susceptibility to tuberculosis in a high-incidence community in South Africa. Int J Tuberc Lung Dis.

[6] Liaw YS, Tsai-Wu JJ, Wu CH, Hung CC, Lee CN, Yang PC (2002). Variations in the NRAMP1 gene and susceptibility of
tuberculosis in Taiwanese. Int J Tuberc Lung Dis.

[7] Zhang W, Shao L, Weng X, Hu Z, Jin A, Chen S (2005). Variants of the natural resistance-associated macrophage protein 1 gene
(NRAMP1) are associated with severe forms of pulmonary tuberculosis. Clin Infect Dis.

[8] Takahashi K, Hasegawa Y, Abe T, Yamamoto T, Nakashima K, Imaizumi K, Shimokata K (2008). SLC11A1 (formerly NRAMP1)
polymorphisms associated with multidrug-resistant tuberculosis. Tuberculosis.

[9] Gao PS (2000). Genetic variants of NRAMP1 and active tuberculosis in Japanese populations. International Tuberculosis Genetics
Team. Clin Genet.

[10] Liu W, Cao WC, Zhang CY, Tian L, Wu XM, Habbema JD (2004). VDR and NRAMP1 gene polymorphisms in susceptibility to
pulmonary tuberculosis among the Chinese Han population: a case-control study.. Int J Tuberc Lung Dis.

[11] Ryu S, Park YK, Bai GH, Kim SJ, Park SN, Kang S (2000). 3'UTR polymorphisms in the NRAMP1 gene are associated with
susceptibility to tuberculosis in Koreans.. Int J Tuberc Lung Dis.

[12] Duan HF, Zhou XH, Ma Y, Li CY, Chen XY, Gao WW, Zheng SH (2003). A study on the association of 3'UTR polymorphisms of
NRAMP1 gene with susceptibility to tuberculosis in Hans. Zhonghua Jie He He Hu Xi Za Zhi.

[13] Hatta M, Ratnawate, Tanaka M, Ito J, Shirakawa T, Kawabata M NRAMP1/SLC11A1 gene polymorphisms and host
susceptibility to Mycobacterium tuberculosis and M. leprae in South Sulawesi, Indonesia. Southeast Asian J Trop Med Public
Health.

[14] Medina E, North RJ (1996). Evidence inconsistent with a role for the Bcg gene (Nramp1) in resistance of mice to infection with virulent
Mycobacterium tuberculosis. J Exp Med.

[15] Kim JH, Lee SY, Lee SH, Sin C, Shim JJ (2003). NRAMP1 genetic polymorphisms as a risk factor of tuberculous pleurisy. Int J Tuberc Lung Dis.

[16] Selvaraj P, Chandra G, Kurian SM, Reetha AM, Charles N, Narayanan PR (2002). NRAMP1 gene polymorphisms in pulmonary and
spinal tuberculosis. Curr Sci.

[17] McDermid JM, Prentice AM (2006). Iron and infection: effects of host iron status and the iron-regulatory genes haptoglobin and
NRAMP1 (SLC11A1) on host-pathogen interactions in tuberculosis and HIV. Clin Sci.

